# Utilization of High-Zn Content Ferrous Landfill Sludge with the Use of Hydrogen

**DOI:** 10.3390/ma16247676

**Published:** 2023-12-16

**Authors:** Mikolaj Bernasowski, Piotr Migas, Marta Ślęzak, Łukasz Gondek, Łukasz Cieniek

**Affiliations:** 1 Faculty of Metals Engineering and Industrial Computer Science, AGH University of Krakow, Mickiewicza 30, 30-059 Krakow, Poland; 2 Faculty of Physics and Applied Computer Science, AGH University of Krakow, Mickiewicza 30, 30-059 Krakow, Poland

**Keywords:** hydrogen, iron oxides reduction, DRI, zinc recovery, sludge utilization

## Abstract

Sludge, due to its form and significant moisture and zinc content, is the most problematic metallurgical waste. Near the site of a disused steelworks plant in Krakow (Poland) there is an estimated 5 million tonnes of landfill sludge that consists of more than 90% iron and other metal oxides. There is a global tendency to switch steel production towards carbonless technologies, which is why the presented work investigates the possibility of simultaneous waste liquidation and recovery of valuable metals with the use of hydrogenous reduction. Direct reduced iron (DRI) production was selected as the targeted technology, so the sludge was lumped and bound with cement or CaO addition. The obtained lumps were reduced in a hydrogenous atmosphere with gradual heating to 950 °C, after which their phase structure was analyzed and elemental analysis was performed. It was found that zinc evaporated during the experiment, but mostly thanks to the carbon contained in the sludge. The increased addition of binder to the sludge resulted in the enhancement of the lumps, but also limited the reduction range. The products obtained were mostly wustite and less pure iron. Taking into account the degree of reduction and the lumps’ compression strength, the best binding was achieved by adding cement at a quantity of 5% mass.

## 1. Introduction

Steel is the second most common man-made material after concrete (annual global steel production is approximately 2 billion tonnes [1,2]). Steel is a material that enables the implementation of green energy technologies because it is infinitely recyclable, and its residues and waste can become valuable resources, thus contributing to a sustainable and circular economy. Directives and policies of the European Union and the Paris Agreement assume achieving climate neutrality by 2050, which necessitates the development of technologies aimed at almost eliminating CO_2_ emissions (reducing CO_2_ emissions from steel production by half by 2030 and 80–95% by 2050) [3]. Steel production accounts for approximately 8% of global carbon dioxide emissions. During steel production, the total CO_2_ emission rate is 1.8 t CO_2_/t steel. The most harmful iron and steel industry installations are the raw materials departments (coking plants, sinter plants, blast furnaces) that are located in integrated steelworks. In coke production, atmospheric pollution is mainly caused by leaks of hard coal distillation products from equipment. In sinter plants, the technological process involves burning coke, and the resulting gases (CO_2_, CO, NO_2_, SO_2_ and others) contain dust. In the blast furnace process, threats to the atmosphere include escaping blast furnace gas, dust from the blast furnace charge, and fumes floating above the hot metal and slag gutters. Steel is produced in basic oxygen furnaces (BOF) or electric arc furnaces (EAF). Both processes produce dust and gas emissions, which also occur in the process of secondary metallurgy in ladle furnaces and, to a small extent, during continuous steel-casting processes. The production of hot metal in a blast furnace emits more greenhouse gases than any other metallurgical process [4,5]. Hydrated dust (sludge) is a waste material originating from blast furnace and converter processes; it contains mainly iron oxides and other reducible oxides, including zinc oxides.

In the European Union, approximately twenty-eight integrated steelworks operate, producing sludge as a waste material from BF and BOF, including precipitating sludge generated by galvanizing processes [6,7]. Taking into account the average amounts of waste generated in these units in Europe in 2021, it can be estimated that the following amounts of this type of sludge were generated: 12 kg/t × 87 million t = 1.044 million t from hot metal production, and 16 kg/t × 93.9 million t = 1.502 million t from steel production. In total, over 2.5 million tonnes of sludge were produced in steelworks in the EU in 2021 [1,6,8,9,10,11,12].

Steel production is based on blast furnace technology that uses iron ore, coke and limestone to produce hot metal, which is then processed into steel in an oxygen converter. In turn, the material input into an electric arc furnace is steel scrap. Due to the amount of scrap available, approximately 30% of the world’s steel is produced in arc furnaces. An alternative steel production technology is the processing of materials by means of the direct reduction of iron ore, namely, DRI (direct reduced iron) [13].

Direct reduction involves the reduction of iron oxides to metallic iron in the solid state, which occurs at temperatures below the melting point of iron in the presence of a reducing synthesis gas, most often natural gas. Modern solutions aim to replace natural gas with hydrogen gas to reduce CO_2_ emissions from this process [14,15,16,17,18,19,20].

At least five research projects are currently being implemented in the European Union that are devoted to the use of hydrogen in metallurgy. The largest undertaking is the HYBRIT Demonstration project, which is a Swedish large-scale steel value chain demonstration of Hydrogen Breakthrough Iron-making Technology [17]. The available information indicates that this project focuses on adapting a DRI reactor to use hydrogen as a reductant.

**Figure 1:**
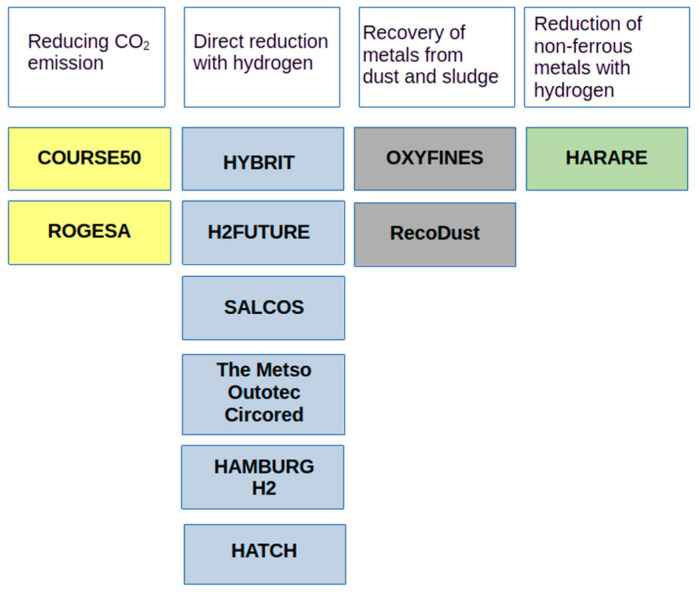
Worldwide projects involved in reducing CO_2_ emissions in steel production.

Figure 1 presents the list of projects implemented around the world related to the issue of reducing CO_2_ emissions, the use of hydrogen as a reductant in ferrous and non-ferrous metallurgy, and the management of metallurgical wastes [14,15,16,17,18,19,20].

The COURSE50 project (Asia, Japan) aims to develop technologies to reduce CO_2_ emissions from blast furnaces by approximately 30% and capturing, separating and recovering CO_2_ from blast furnace gas (BFG).

The ROGESA Roheisengesellschaft Saar mbH (Europe, Dillingen) project is intended to enable the development of a new technology by which coke oven gas will become a gas used in the blast furnace process; this will partially replace metallurgical coke, thus contributing to reducing carbon dioxide emissions from blast furnaces.

The HYBRIT (Europe, Scandinavia) initiative was launched in 2016 at Vattenfall by the Swedish steel company SSAB (Stockholm, Sweden) and a Swedish mining company LKAB (Lulea, Sweden). The project concerns the direct reduction of iron ore with hydrogen obtained from water electrolysis, with the goal of reducing CO_2_ emissions by 10% in Sweden and 7% in Finland. 

The H2FUTURE (Europe) project, initiated by Voestalpine in 2017, aims to reduce CO_2_ emissions from steel production by developing a breakthrough technology that could replace coke with hydrogen, thus ultimately reducing CO_2_ emissions by 80% by 2050.

Salzgitter AG’s (Europe) SALCOS project began in 2019. It investigates the use of hydrogen as a reducing agent in iron production to reduce CO_2_ emissions. By using hydrogen, this project aims to gradually transform the conventional and high-emission blast furnace process into a DRI direct reduction process. 

The Metso Outotec Circored (South America) process is a 100% hydrogen-based direct reduction process that produces highly metalized sponge iron (DRI) or hot briquetted iron (HBI) that can be fed directly into an electric arc furnace.

In turn, the OXYFINES project (Europe, Sweden) concerns the developed OXYFINES technique, which is suitable for recovering zinc from metallurgical waste, including sludge. 

Hamburg H2 (Europe) started a project at the ArcelorMittal plant in Hamburg, Germany, with the main goal of determining whether it is possible to replace natural gas with hydrogen to reduce iron ore and perform DRI with the use of H_2_ on an industrial scale; the secondary goal is to test how this carbon-free DRI reacts in an EAF [21].

Hatch (Europe) is supporting Tata Steel in the Netherlands in a project that aims to develop hydrogen-based steel production. As part of this project, Hatch’s CRISP+ furnace technology will be developed to produce green steel as part of Tata Steel’s strategy to produce steel with zero carbon emissions by 2045 [22].

The RecoDust project (Europe, Austria) is a pyrometallurgical approach to the processing of metallurgical dust containing heavy metals that provides two products: iron-rich RecoDust slag and zinc-rich filter dust, called crude zinc oxide.

The HARARE (Europe) consortium consists of ten industrial and research partners from four European countries. The project aims to develop a sustainable path for the production of non-ferrous metals using hydrogen as a factor that enables waste removal and the valorization of materials in emission-free processes.

The issue of zinc recovery from sludge and process dust was the subject of a publication [23,24,25,26] in which the authors [23] provided a method of removing zinc from sludge by multilayer sintering. Dezincification occurs thanks to additional carbonous reductants derived from an additional layer of coke breeze that is directly placed on the sludge layer.

Another paper [24] studied the possibility of reducing zinc from an EAF dust sample through an H_2_ constant flux in a horizontal oven. The tests show that the reduction process is thermodynamically viable for temperatures higher than 850 °C, and all zinc metal produced is transferred to the gas stream, enabling its complete separation from iron. 

In the paper [25], the authors conducted experimental studies on the use of hydrogen as a reducing agent for zinc ferrite in EAF dust. They realized that the reduction of zinc ferrite in EAF dust using hydrogen was promoted by increasing the reaction temperature from 500 to 800 °C. Zinc vapor and metallic iron were produced at temperatures above 700 °C. It is stated that hydrogen could be used to extract zinc and iron from zinc ferrite at temperatures in the range of 700 to 800 °C.

Other authors [26] confirmed the possibility of using hydrogen as a reducing agent. They stated that the main advantage of hydrogen is increased chemical kinetics, which allows for lower process temperatures, which in turn leads to reduced heat loss and equipment wear.

For over 3000 years, carbon has been used as a reducing agent in the reduction of metal oxides. The reduction mechanism as well as the kinetics and thermodynamics of the iron and zinc oxide reduction process have been the subject of numerous studies and publications [27,28,29,30,31,32].

Currently, hydrogen is being considered as a new reductant of metal oxide. The thermodynamic basis of the reduction of iron oxides with hydrogen is known, and the equilibrium constants in the state of thermodynamic equilibrium of the system have been investigated. The degree of reduction usually increases with increasing temperature, hydrogen content, and pressure [33,34,35,36,37].

The reduction of iron oxides with hydrogen, depending on the process temperature, takes place in two or three stages [14].

Regardless of the temperature at which the reduction process takes place, the first stage of reduction is the same and involves the reduction from hematite to magnetite (1):

(1) $$3{Fe}_{2}O_{3}+H_{2}\to 2{Fe}_{3}O_{4}+H_{2}O,$$

The second stage depends on the temperature of the reduction process. At temperatures below 570 °C, only the second stage of the reduction reaction occurs, which involves the reduction of magnetite directly to iron (2):

(2) $${Fe}_{3}O_{4}+{4H}_{2}\to 3Fe+{4H}_{2}O,$$

If the reduction process takes place at a temperature above 570 °C, the reduction involves a total of three stages, the second and third of which are described by the equations for the reduction of magnetite to wustite (3) and wustite to iron (4):

(3) $${Fe}_{3}O_{4}+H_{2}\to 3FeO+H_{2}O,$$

(4) $$FeO+H_{2}\to Fe+H_{2}O,$$

The reduction of zinc ferrite takes place as follows [25,26]:

(5) $$Zn{Fe}_{2}O_{4}+4CO\to Zn+2Fe+4CO_{2},$$

(6) $$Zn{Fe}_{2}O_{4}+{4H}_{2}\to Zn+2Fe+4H_{2}O,$$

In works [38,39,40,41], the authors researched the direct reduction of iron ore using hydrogen. Synthetic hematite samples were reduced using a mixture of pure hydrogen with 10% helium in the 550–900 °C temperature range [38]. The gas flow rate was set to 70 cm^3^/min. The total conversion time was examined from 350 s to approximately 1100 s at 800 °C. It was observed that the reduction of the hematite samples was a multi-stage reaction with one or two intermediate oxides, depending on temperature. The provided analysis shows that the longest step is the transformation from wustite to iron. The transformations from hematite to magnetite and magnetite to wustite were successive and well separated, since the hematite completely disappeared when the first grains of wustite were detected. It is interesting that the reduction of wustite into metallic iron begins before the total consumption of magnetite. Experiments with three types of 99.9% pure hematite powder samples have shown differences in reactivity: a coarse sample with grains smaller than 5 μm was more reactive than samples with grains of about 1 μm in diameter; the authors called these very fine grains “nanopowder”.

Hematite ore samples and hydrogen have also been examined as a reducing agent [39]. The temperature range was between 500 and 900 °C. The sample amount varied between 80, 100, and 140 g, with an average particle size of 69 µm. The effect of reaction time on the reducibility of the hematite sample was demonstrated, and the results show an optimum reaction time of 30 min. In the described experiments, the reduction rate increased until a temperature of 750 °C was reached. As the temperature was further increased, the reduction rate started falling slightly and rose again as the temperature increased over 850 °C. It was found that the optimal amount of hematite for these authors’ lab conditions was 80 g, with a pure hydrogen flow rate of 5 L/min, temperature of 750 °C, and reduction time of 30 min. In these conditions, the reduction rate was around 90%. For the heavier sample (about 140 g), the reduction rate was 10% lower.

The authors of [40] used pellets made from iron ore powder, pine sawdust, and bentonite (96%, 2%, and 2%, respectively). Particle sizes of 10–15 mm were taken as the testing samples. The roasted pellets were reduced in the temperature range 850 °C to 1050 °C with pure hydrogen (gas flow rate was 7 cm^3^/s). The authors observed that the reduction in temperature had a significant effect on the extent of the reduction. At the same reduction temperature, the reduction of pellets containing biomass was relatively higher than for those without biomass, because it increased the porosity of the pellets.

Hematite powder with an average particle size of 74 µm has been reduced by pure hydrogen with a flow of 3 L/min in a temperature range of 600 to 1050 °C [41]. It was observed that the reduction rate linearly increased from 600 to 1000 °C. On the other hand, the reduction rate decreased above 1000 °C due to sintering, which causes a decrease in porosity and the formation of a denser structure. It was also observed that the reduction from wustite to metallic iron is the slowest step of the whole reduction process. 

Assuming the results presented above, landfilled metallurgical wastes contain valuable metals, but their extraction can be problematic technologically or environmentally. This sludge, which is usually very wet and has an unstable chemical composition, is hard to deal with. Zinc has to be separated before reduction processes in a blast furnace—still the main process in iron production—and the costs of energy-intensive drying are high. Taking into account the gradually growing interest in hydrogen reduction technologies, it was decided to combine the needs of metal recovery with environmentally friendly technology; therefore, the presented study attempts to use hydrogen as a reductant in sludge utilization.

## 2. Materials and Methods

Because the sludge was extremely wet, it was decided to mix it with calcium-bearing binder in order to form spherical lumps, thus making it similar to the ore pellets that are used as a burden in the DRI process.

**Figure 2:**
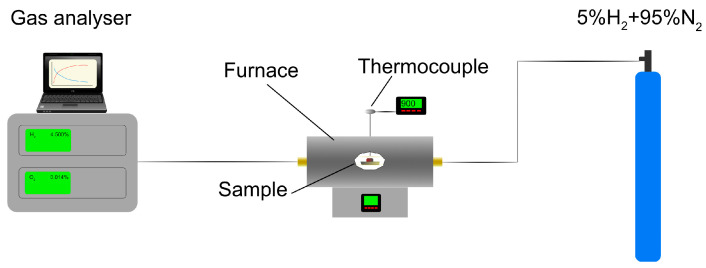
Schema of equipment used for reduction processes.

A 30 kg sample of sludge was taken from a random place at the metallurgical waste landfill near Krakow’s disused steel mill. The material was mixed with two types of binder, CaO and Portland cement, in proportions 5, 10 and 15 mass%. This caused lumps of approximately 20 mm diameter to be formed. After waiting 7 days, the compressive strength of the samples was examined, after which they were exposed to a reducing atmosphere of 5%H_2_ + 95%N_2_ with 80 L/h gas flow in a horizontal tube furnace with a gradual increase in temperature up to 950 °C. This maximum temperature was chosen to prevent excessive softening and the lumps sticking to each other. The H_2_ concentration in the flue gas was constantly monitored and recorded. The average heating rate of the furnace was 8.5 °C/min. The samples were then cooled in a N_2_ atmosphere. The general schema of equipment for this reduction process is shown in Figure 2. 

The raw material and the lumps were examined after reduction.

X-ray diffraction (XRD) patterns were collected in the standard Bragg–Brentano geometry. A Malvern Panalytical Empyrean diffractometer (MalvernPanalytical, Almelo, The Netherlands) equipped with Cu Kα radiation source was used. The diffractometer was calibrated for geometry and reflection profiles with a LaB6 sample (NIST 660).

The chemical composition of the selected material was analyzed using an FEI Nova NanoSEM 450 high-resolution scanning microscope (Hillsboro, OR, USA) equipped with an Octane Elect Plus energy-dispersive detector (EDS) based on SDD (Silicon Drift Detector, AMETEK, Inc., Berwyn, IL, USA) technology, with high-speed electronics and specialized APEX(version 2.2.0001.0001)/GENESIS (version 6.34) software from EDAX. Samples underwent an initial examination using ETD/TLD for secondary electron contrast and a CBS ring detector for backscattered imaging. This allowed for the preliminary selection of sites and subsequent quantitative and qualitative chemical composition analysis.

Due to the elements’ complex composition, variable current–voltage parameters were utilized (as given in ranges) for further analysis:

- The accelerating voltage used was 10–15 kV;
- The beam current (spot) range was 3–4;
- The working distance (WD) was approximately 3.4–6.0 mm, individually selected for each sample;
- The aperture size was 4–7.

All these operating parameters allowed the characteristic X-rays of the analyzed elements to be excited. The following parameters were obtained for X-ray detection using a solid-state EDS detector (AMETEK, Inc., Berwyn, IL, USA):

- The number of counts per second (Cps) was approximately 1000–2500;
- The dead time (DT) was approximately 6–20%;
- The time constant was 12.8 μs.

## 3. Results and Discussion

### 3.1. Preliminary and High-Temperature Research

**Figure 3:**
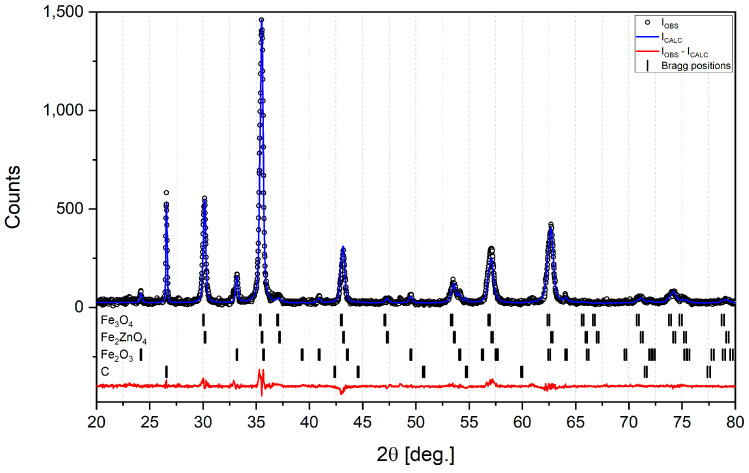
XRD analysis with Bragg positions for raw sludge.

**Figure A1:**
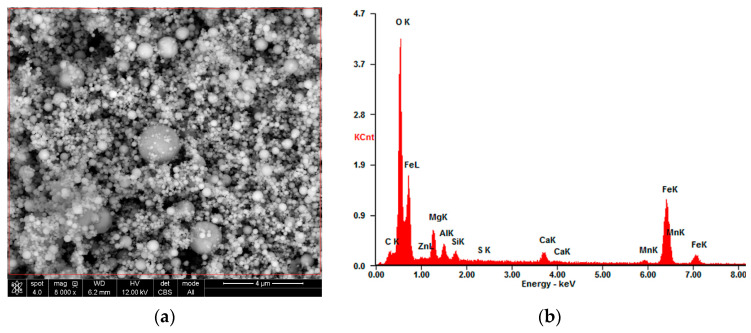
Raw sludge analysis: (**a**) high-magnification SEM image and scanning area; (**b**) EDS spectra of inclusions in the area marked by the red box.

Table 1 shows the phase composition of raw sludge determined by means of XRD analysis; Bragg positions are shown in Figure 3. It can be seen that there is a high content of iron zinc oxide (Fe_2_ZnO_4_), which translates into almost 16% of zinc. Even though waste from the Krakow steelworks usually contains about 3% Zn, this high value can possibly be explained by the fact that some of this landfill waste came from the galvanizing plant in a cold-rolling mill in the 1990s. It should be noted that XRD analysis did not detect any significant content of substances such as silica, alumina or calcium oxide. Also, the results of the SEM/EDS elemental analysis of raw sludge show very low contents of Si, Al and Ca (See Appendix A, Figure A1a,b and Table A1). Such a low content of these substances could be explained by washing them away from the upper layers of the sludge during long-term landfilling. So, the diversity in the sludge’s chemical composition of zinc and other elements must be continuously monitored during its possible future utilization.

Table 2 presents the compression strength of the lumps containing binders in different proportions and masses before and after reduction in the hydrogenous atmosphere. It can be seen that adding both types of binder also increased the strength of the lumps, but those bound with cement were more durable than those bound with CaO. After obtaining the XRD data, it was presumed that the binder slows the reduction process; therefore, lumps with the addition of 5% binder were considered the most promising from the perspective of metal recovery.

Additionally, it was decided to make lumps from pure oxides Fe_2_O_3_:Fe_3_O_4_:ZnO in the proportion 50:30:20, with an admixture of 5% cement or CaO but without carbon (as was detected in the sludge), in order to exclude the influence of the latter on the metal reduction. These synthetic lumps without carbon are called Synthetic Cement5% or Synthetic CaO5%. 

**Figure 4:**
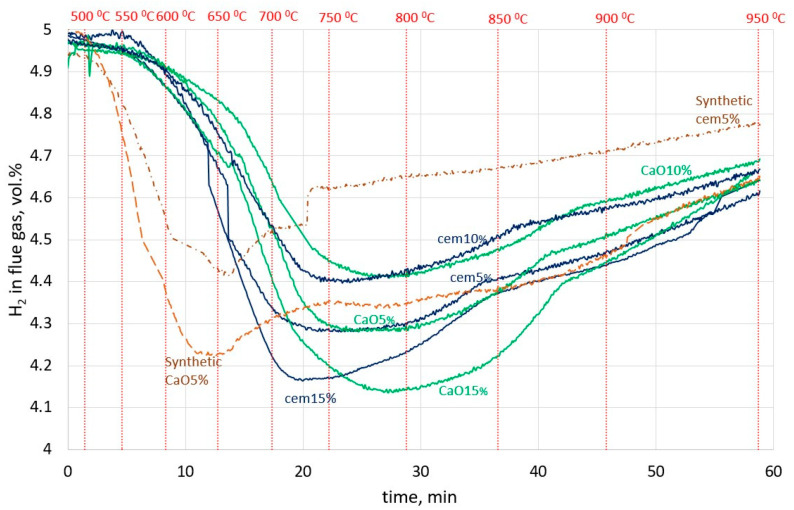
H_2_ change over time in the flue gas in the experiment for all types of lumps.

Figure 4 presents the H_2_ change over time in the flue gas during the reduction of lumps in the furnace. All the sludge lumps started to reduce at over 550 °C, but the synthetic lumps started to reduce sooner, at a temperature of about 500 °C. This could be caused by the presence of pure oxides and the absence of complex oxide Fe_2_ZnO_4_, which constitutes the largest part of the sludge. It can be seen that the curves of all cement sludge lumps reached a minimum H_2_ content in flue gas earlier than the corresponding CaO sludge lumps. This can be explained by the faster reduction in lumps containing cement binder. So, it seems that the CaO binder makes the reduction processes slower. 

### 3.2. XRD Analysis of All Lumps after Reduction

**Figure 5:**
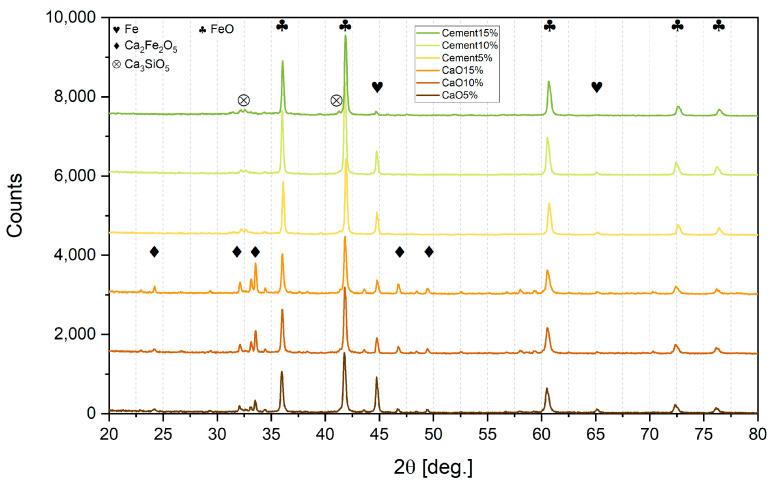
XRD analysis of sludge lumps.

**Figure 6:**
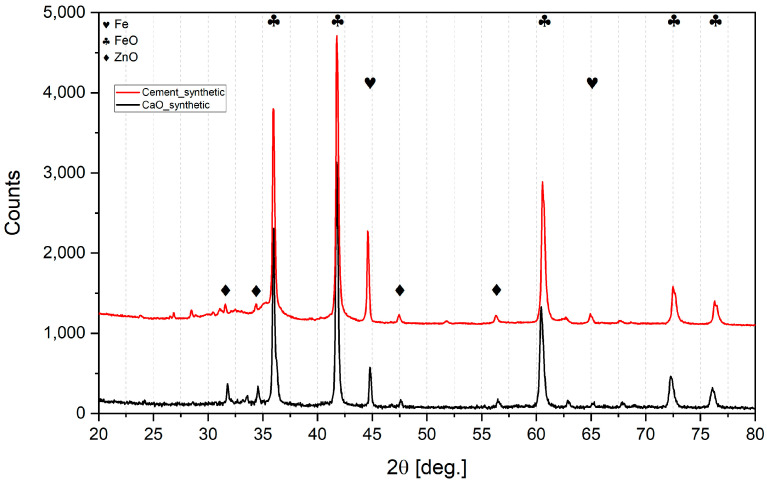
XRD analysis of synthetic lumps.

Table 3 and Figure 5 and Figure 6 present the results of XRD analysis and calculated metallization and reduction degrees of all the lumps after reduction. 

Based on XRD analysis, the metallization degree (MD) and reduction degree (RD) were calculated according to Equations (7) and (8), respectively, as shown in Table 4.

(7) $$MD=\frac{{Fe}_{met}}{{Fe}_{sum}}\cdot 100,$$

(8) $$RD=\frac{O_{sludge}-O_{lump}}{O_{sludge}}\cdot 100,$$

where:

Fe_met—_metallic iron in lumps after reduction, %;
Fe_sum_—the sum of metallic iron and iron in oxides in lumps after reduction, %;
O_sludge_—oxygen in iron and zinc oxides in sludge before reduction, %;
O_lump_—oxygen in iron and zinc oxides in lumps after reduction, %.

**Table 4:** Metallization and reduction degrees calculated by means of XRD analysis of lumps.

Parameter	Cement5%	Cement10%	Cement15%	CaO5%	CaO10%	CaO15%	Synthetic Cement5%	Synthetic CaO5%
Metallization degree, %	15.5	9.7	3.4	20.8	14.5	6.3	10.9	12.5
Reduction degree, %	41.9	40.2	39.4	31.5	27.8	23.7	38.9	26.4

It can be seen from Table 4 that the increased addition of both types of binder to the sludge gradually decreased the reduction range. With every 5% of binder addition, the metallization degree dropped significantly. So, a 5% addition of binder is most effective for reduction. 

It also can be observed that lumps bound with CaO contained more pure iron and less wustite than lumps bound with cement in respective proportion admixtures. However, calcium ferrite Ca_2_Fe_2_O_5_ was detected in CaO lumps, so CaO addition may bind hematite into a compound that is difficult to reduce under these experimental conditions. Previous authors [42] used a much more reducible atmosphere, and determined that the reduction of calcium ferrites in sinter can only occur after the hematite is exhausted; moreover, depending on the temperature, it can take 60 min at 500 °C or 30 min at 700 °C. 

On the other hand, CaO is bound to SiO_2_ in cement lumps due to the pozzolanic binding processes, and this is possibly why the reduction degree is much higher than in CaO lumps.

In all sludge lumps, the XRD analysis did not indicate the presence of zinc. Presumably, the reason for this was the presence of carbon in the sludge, even a small amount of which significantly enhances the reducing capability in the reaction phase. In an attempt to prove this, lumps were made of pure iron and zinc oxides. Even though the zinc oxide was pure and did not present Fe_2_ZnO_4_ as in the sludge, a significant amount of ZnO remained after the hydrogen reduction both when using CaO and when using cement as binders. So, it is likely that the presence of even small amounts of carbon facilitates the reduction, processes and is crucial to ensure zinc recovery. In the presence of carbon, zinc oxide can be reduced directly in two stages ((9) + (10) = (11)):

ZnO + H_2_ = Zn + H_2_O, (9)

H_2_O + C = CO + H_2_ (10)

ZnO + C = Zn + CO(11)

Assuming the presented binders and their admixture proportion, and taking into account the lumps’ strength, metallization, and reduction degree, making sludge lumps by binding them with a 5% addition of cement can be selected as the most optimal way to prepare a burden for the DRI process. Thus, these lumps were subjected to SEM analysis. 

### 3.3. SEM Analysis of Raw Sludge and 5% Binder Admixed Lumps

**Figure 7:**
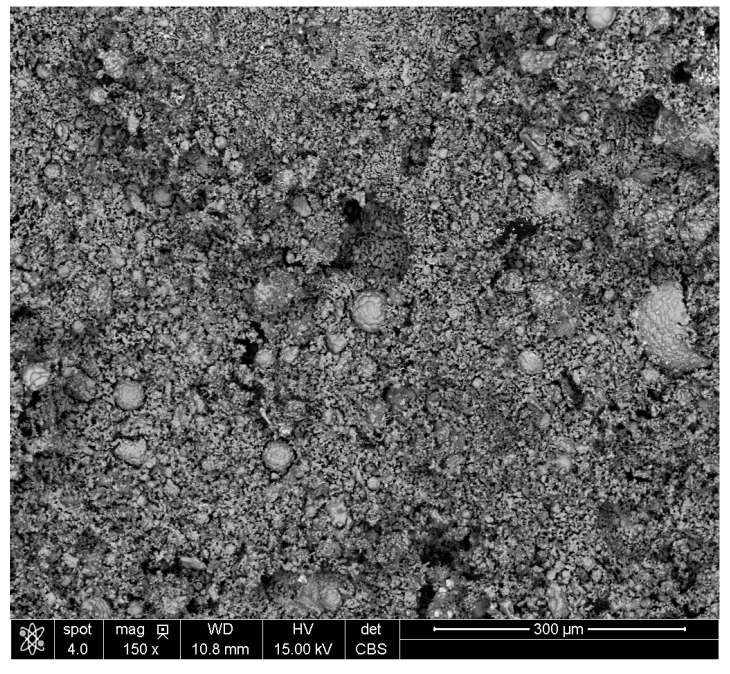
Macro view of the Cement5% lump after reduction.

**Figure A2:**
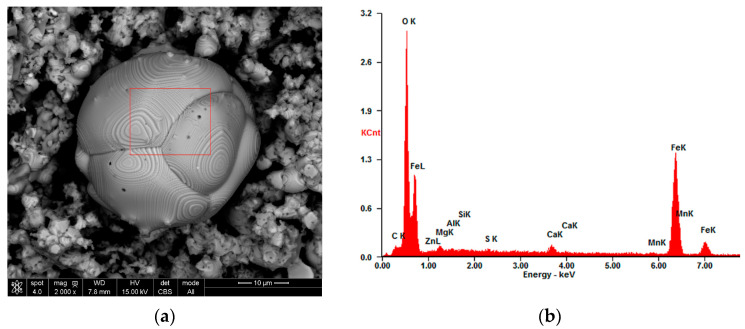
Nodule analysis: (**a**) high-magnification SEM image and scanning area; (**b**) EDS spectra of inclusions in the area marked by the red box.

Figure 7 shows a macro view of a 5% cement lump after reduction. At 150× magnification, large nodules can be observed, which were identified as wustite (See Appendix A, Figure A2a,b and Table A2). 

**Figure 8:**
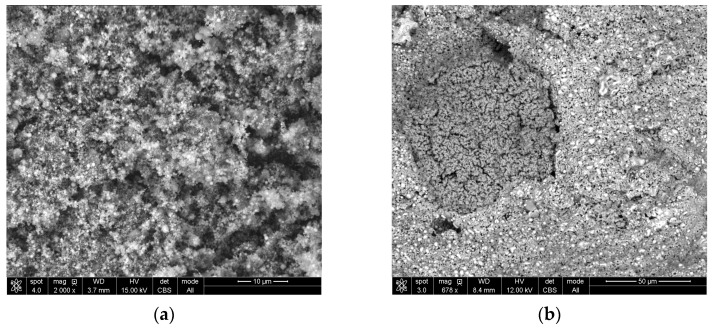
Macro view of: (**a**) raw sludge; (**b**) lump bound with 5% CaO.

It was decided to also make macro views of raw sludge and a CaO5% lump after reduction, as are shown in Figure 8a,b, respectively. In sludge, particles are so small that they can only be seen at 2000× magnification; however, in CaO5% lumps, the structure is homogeneous and has pores without nodules.

**Figure 9:**
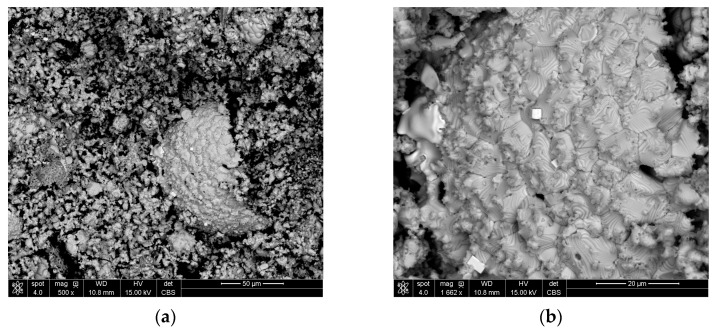
Wustite nodule in the structure of Cement5% with cubes of pure iron at magnification (**a**) 500× and (**b**) 1662×.

**Figure A3:**
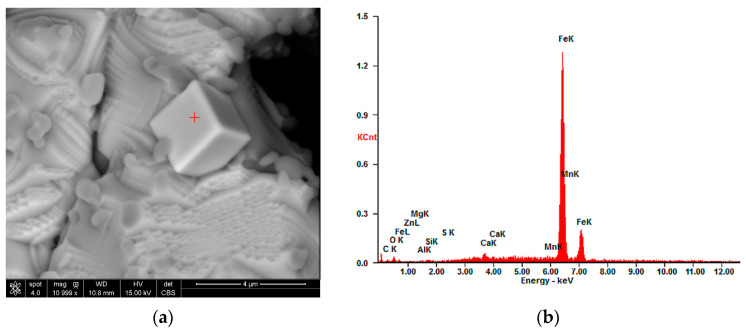
Bright cube analysis: (**a**) high-magnification SEM image and scanning area; (**b**) EDS spectra of inclusions in point marked with the red plus.

The occurrence of nodules can be explained by the addition of cement as a binder to lumps, because the coagulation of iron oxide particles occurs during cement binding, which probably ensures the strength of the lumps. These particles remain in clusters, and after reduction, turn into nodules. Figure 9 shows a wustite nodule at magnifications of 500× (a) and 1662× (b). Bright cubes can be observed on the surface of the nodule. These cubes were identified as pure iron (See Appendix A, Figure A3a,b and Table A3). This confirms that even such a low concentration of the reductant in the gas provides conditions for the reduction of iron from wustite. However, the nodules’ presence could be responsible for the lower degree of metallization.

### 3.4. Future Directions of Sludge Utilization Research

The main finding of the presented work is the method of sludge preparation as a burden for use in the DRI process. However, some aspects should be investigated.

Firstly, the metallization degree is not satisfactory for DRI production. So, further tests should focus on increasing the hydrogen concentration in the gas or extending the time of the lumps’ exposure to hydrogen. However, it is hard to achieve maximum metallization efficiency in these lumps even when using pure hydrogen [43]. The time of reduction could also be crucial, especially when reduction proceeds in the narrow temperature range of ~550–950 °C.

**Figure A4:**
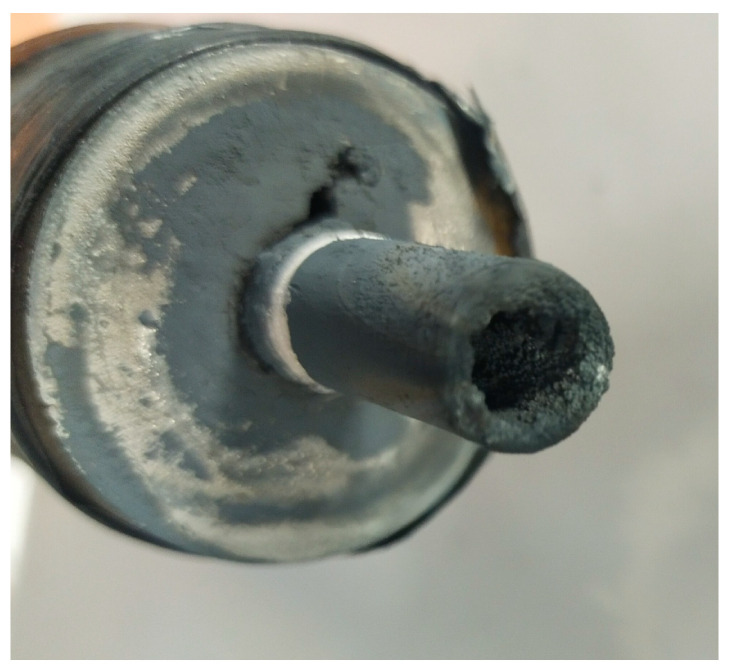
Lab furnace outlet manifold with zinc-bearing deposit (the photo was taken after completing all tests).

Secondly, the diversity of the zinc concentration in the sludge should be considered. Sludge with high zinc concentrations should be considered for processing in zinc-recovery devices like the Waelz kiln. On the other hand, full zinc recovery was obtained in the presented study. A deposit consisting of metallic zinc and zinc oxide was observed on the furnace outlet manifold (see Appendix A, Figure A4), thus suggesting that the reduction device could be customized for the collection of zinc-bearing concentrates.

## 4. Conclusions

A wet form of sludge encourages the formation of lumps with the addition of a CaO-bearing binder. The addition of cement seems to be more favorable.

The utilization of sludge by means of hydrogen does not mean zero CO_2_ emissions, as this sludge contains some carbon. This carbon is very helpful in sludge dezincification because it is hard to fully reduce both iron and zinc using hydrogen only.

In Cement5% lumps, big nodules of wustite are visible; however, wustite occurs more evenly throughout the cross-section in CaO5% lumps. On the other hand, after reduction, Cement5% had a lower metallization degree than CaO5% lumps. It seems that the large, tight formations of wustite slow down the iron reduction.

## Figures and Tables

**Table 1:** Phase composition of raw sludge.

	Fe_2_ZnO_4_ ^(on dry)^	Fe_3_O_4_ ^(on dry)^	Fe_2_O_3_ ^(on dry)^	C ^(on dry)^	Moisture
Raw sludge, %	58.26	29.60	10.45	1.69	38.32

**Table 2:** Compression strength and masses before and after the reduction process for all kinds of lumps.

Parameter	Cement5%	Cement10%	Cement15%	CaO5%	CaO10%	CaO15%	Synthetic Cement5%	Synthetic CaO5%
Compression strength, N/psc	120	140	175	70	136	140	115	70
Sample mass, g	15.41	15.00	16.50	15.72	17.28	16.15	13.97	14.67
Mass after reduction, g	11.43	11.99	13.90	12.12	13.83	12.40	11.37	12.17
Weight loss, g	3.98	3.01	2.60	3.60	3.45	3.75	2.60	2.50
Absolute weight change, %	25.82	20.06	15.75	22.90	19.97	23.22	18.61	17.04

**Table 3:** Detected phase share in XRD analysis after reduction.

Detected Phase	Cement5%	Cement10%	Cement15%	CaO5%	CaO10%	CaO15%	Synthetic Cement5%	Synthetic CaO5%
Ca_2_Fe_2_O_5_, %	-	-	-	25	30	41	-	23
Ca_3_SiO_5_, %	20	22	25	-	-	-	19	-
FeO, %	70	72	73	60	60	55	63	60
Fe, %	10	6	2	15	10	4	6	8
ZnO, %	-	-	-	-	-	-	12	9

## Data Availability

Data are contained within the article.

## Appendix A

**Table A1:** SEM/EDS results for raw sludge.

Element	CK	OK	ZnL	MgK	AlK	SiK	SK	CaK	MnK	FeK
Wt. %	5.0	33.6	5.5	0.0	0.7	0.3	0.0	0.9	0.0	54.0
At%	11.5	57.9	2.3	0.0	0.7	0.3	0.0	0.6	0.0	26.7

**Table A2:** SEM/EDS results for nodule.

Element	CK	OK	ZnL	MgK	AlK	SiK	SK	CaK	MnK	FeK
Wt. %	3.1	19.7	0.4	0.9	0.4	0.2	0.3	1.4	1.1	72.4
At%	8.9	42.4	0.2	1.3	0.5	0.2	0.3	1.2	0.7	44.4

**Table A3:** SEM/EDS results for cube.

Element	CK	OK	ZnL	MgK	AlK	SiK	SK	CaK	MnK	FeK
Wt. %	0.2	0.3	0.0	0.0	0.0	0.1	0.00	1.1	1.4	96.8
At%	1.1	1.0	0.0	0.0	0.0	0.2	0.00	1.5	1.4	94.7
